# In Vitro Reactivity of the Glucose Degradation Product 3,4-Dideoxyglucosone-3-ene (3,4-DGE) towards Abundant Components of the Human Blood Circulatory System

**DOI:** 10.3390/ijms23094557

**Published:** 2022-04-20

**Authors:** Andrea Auditore, Sabrina Gensberger-Reigl, Monika Pischetsrieder

**Affiliations:** Food Chemistry, Department of Chemistry and Pharmacy, Friedrich-Alexander-Universität Erlangen-Nürnberg (FAU), Nikolaus-Fiebiger-Straße 10, 91058 Erlangen, Germany; andrea.auditore@fau.de (A.A.); sabrina.gensberger@fau.de (S.G.-R.)

**Keywords:** 3,4-dideoxyglucosone-3-ene (3,4-DGE), α,β-unsaturated dicarbonyl, glucose degradation product, diabetes, glycation, human serum albumin, l-glutathione, immunoglobulin G, collagen type IV, oxidative stress

## Abstract

3,4-Dideoxyglucosone-3-ene (3,4-DGE) is a glucose degradation product present in processed foods and medicinal products. Additionally, its constant formation from 3-deoxyglucosone in plasma has been suggested. Due to its α,β-unsaturated dicarbonyl moiety, 3,4-DGE is highly reactive and has shown harmful effects in vitro. Here, we investigated the impact of major components of the human blood circulatory system on 3,4-DGE in vitro. Under physiological conditions, plasma concentrations of human serum albumin (HSA) reacted efficiently with 3,4-DGE, resulting in only 8.5% of the initial 3,4-DGE concentration after seven hours (vs. 83.4% without HSA, *p* < 0.001). Thereby, accessible thiol groups were reduced from 0.121 to 0.064 mol/mol HSA, whereas ketoprofen binding and esterase-like activity of HSA were not affected. Plasma concentrations of glutathione (GSH) reacted immediately and completely with 3,4-DGE, leading to two stereoisomeric adducts. Plasma concentrations of immunoglobulin G (IgG) bound to 3,4-DGE to a lower extent, resulting in 62.6% 3,4-DGE after seven hours (vs. 82.2% in the control, *p* < 0.01). Immobilized human collagen type IV did not alter 3,4-DGE concentrations. The results indicated that particularly HSA, GSH, and IgG readily scavenge 3,4-DGE after its appearance in the blood stream, which may be associated with a reduced antioxidative and cytoprotective activity for the living cells and, thus, the human organism by blocking free thiol groups.

## 1. Introduction

Heat treatment and storage of glucose-containing foods [1,2] and medicinal products [3] can lead to the formation of glucose degradation products (GDPs). This heterogeneous class of chemical substances includes mono- and dicarbonyl compounds. Certain GDPs can also be formed in vivo from intermediates of metabolic pathways, such as the polyol pathway [4] or glycolysis [5]. In diabetes mellitus, the elevated glucose concentrations enhance the polyol pathway, contributing to higher GDP-levels under hyperglycemia [4]. The GDP 3,4-dideoxyglucosone-3-ene (3,4-DGE) is of special toxicological concern. The 3,4-DGE is formed by dehydration of 3-deoxyglucosone (3-DG) [6]. It possesses an α,β-unsaturated dicarbonyl structure and, thus, a Michael system, which is mainly responsible for the high reactivity and glycation potential of 3,4-DGE. Previous studies have shown that it is especially susceptible to attacks by free thiol groups of cysteine residues [7,8]. Several harmful effects of 3,4-DGE have been observed in vitro, e.g., cytotoxicity against cell types, such as human podocytes [9] and leukocytes and especially neutrophils, which are involved in host defense against pathogens [10] but also against murine fibroblasts [11]. Possible reasons for the cytotoxicity of 3,4-DGE might be the depletion of cellular l-glutathione (GSH) [12] and, as reported lately, the induction of the nuclear factor-kappa B (NF-κB) pathway and production of reactive oxygen species (ROS) in human keratinocytes [13]. Additionally, impairment of enzyme activity has been described for bovine ribonuclease (RNase) A [11]. Recently, the activation of nociceptors in murine skin and the peritoneum has been observed, which is associated with pain under hyperglycemic conditions [14]. Peritoneal demesothelization is a further serious consequence detected in vitro [15]. Kato et al. have observed reduced proliferation and production of certain murine immune cells in vitro and immunosuppressive effects in vivo [16]. These results suggest, together with the above-mentioned findings by Catalan et al. [10], that 3,4-DGE might increase the risk of severe infections. Moreover, 3,4-DGE can be regarded as a precursor of advanced glycation end products (AGEs) [7]. AGEs are formed during the reaction of reducing sugars or the more reactive GDPs with amino acids and proteins, which is also known as the Maillard reaction. The formation of AGEs has been associated with various diseases, including diabetes, nephropathy, and inflammation [17]. Upon degradation, AGEs can in turn release reactive GDPs [5].

Known sources of 3,4-DGE are foods such as seaweed [16], honey [18], beer (with contents up to ca. 33 µM in malt beer) [19], high-fructose corn syrup [20], and carbonated soft drinks [21]. Considerably higher contents have been found in heat-treated medicinal products containing glucose, mainly in peritoneal dialysis solutions (up to 125 µM directly after sterilization) [22] and infusion fluids (up to 59 ± 1.2 µM) [23]. During the application of infusion fluids, 3,4-DGE is directly injected into the blood circulation, where it may interact with systemic targets. To date, the resorption of 3,4-DGE from food and peritoneal dialysis fluids is not clear. Erixon et al. have not detected 3,4-DGE in human plasma during peritoneal dialysis. However, the plasma levels of 3,4-DGE could have been below the limit of detection of 1.4 µM of the applied method [24], or 3,4-DGE could have reacted quickly with different components in the plasma. Additionally, other products containing glucose or its precursor, 3-DG, could form 3,4-DGE.

Another potential source of 3,4-DGE in the human blood could be a constant transformation from 3-DG. Previous studies have shown that 3-DG, the precursor of 3,4-DGE, is found in the serum of type 2 diabetes mellitus (T2DM) patients in higher concentrations (up to 2.2 µM 3-DG) than in healthy controls [25]. Recently, it has been observed that 3-DG can react to 3,4-DGE in vitro under physiological conditions [14], which indicates a possible formation pathway in vivo. After 24 h, the 3,4-DGE concentration corresponded to 0.6% of the original 3-DG concentration [14]. Thus, a constant formation of about 13 nM 3,4-DGE from 2.2 µM 3-DG in T2DM can be calculated. Especially in diabetic patients, this might represent an additional way of exposure to 3,4-DGE because of their significantly higher 3-DG concentrations compared to healthy controls. In view of the internal and external load of 3,4-DGE, the current study intended to elucidate possible interactions of 3,4-DGE with abundant components of the human blood circulatory system, which are involved in the regulation of oxidative stress, transport of molecules, detoxification, immune system, and vessel stability. The subsequent quantitative analysis of non-bound 3,4-DGE aimed to assess the reactivity of 3,4-DGE towards the applied substrates, namely human serum albumin (HSA), GSH, immunoglobulin G (IgG), and immobilized collagen and to consider the structural and functional consequences of these reactions.

## 2. Results and Discussion

The GDP 3,4-DGE reacted with plasma concentrations of HSA, GSH, IgG, and immobilized collagen. The reactivity towards each of these substrates was determined by quantifying non-bound 3,4-DGE after incubation under physiological conditions.

### 2.1. Reactivity of 3,4-Dideoxyglucosone-3-ene towards Human Serum Albumin

**Figure 1:**
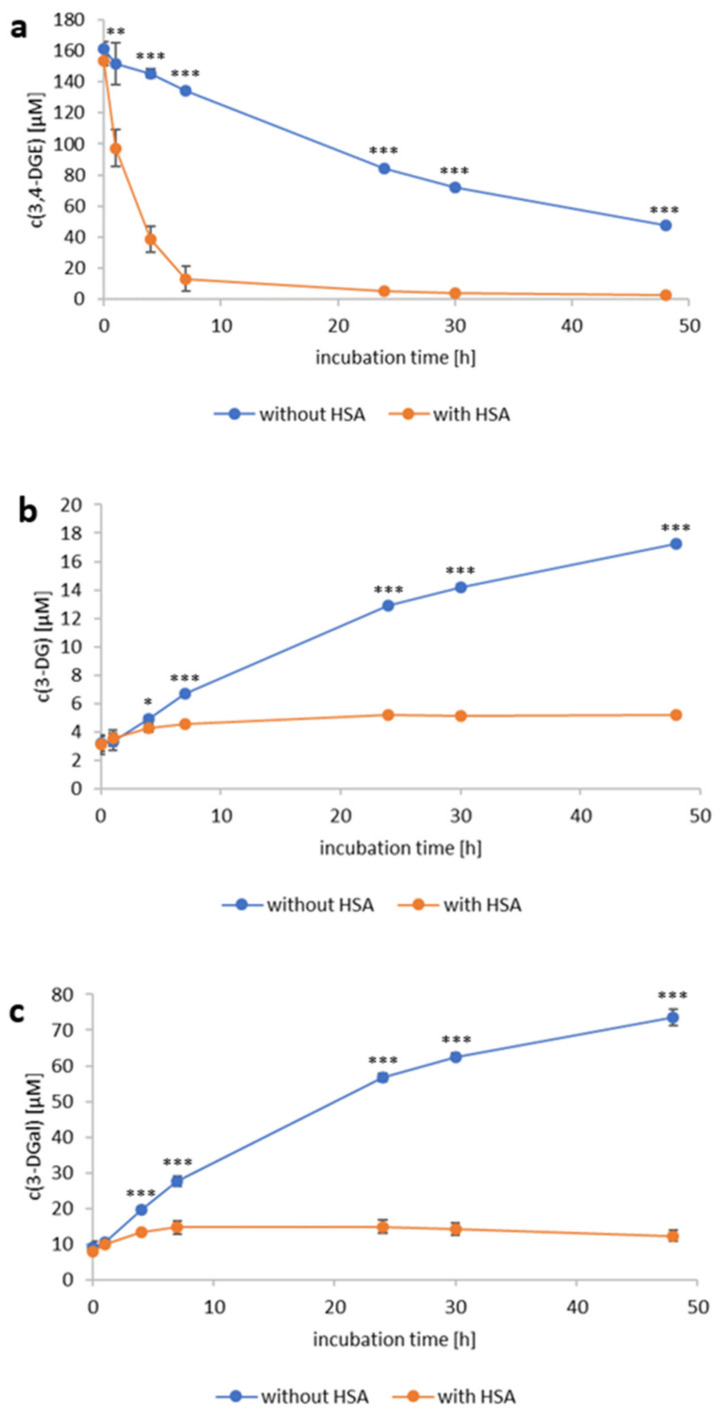
Human serum albumin (HSA) partially depletes 3,4-dideoxyglucosone-3-ene (3,4-DGE) from the model solution and, therefore, reduces its interconversion to 3-deoxyglucosone (3-DG) and 3-deoxygalactosone (3-DGal). 3,4-DGE (161.5 µM, containing 3.2 µM 3-DG and 9.1 µM 3-DGal) was incubated for 48 h at 37 °C in the absence (blue curve) or presence (orange curve) of HSA (40.0 mg/mL). The time course curves for the concentrations (in µM, mean ± SD) of (**a**) 3,4-DGE, (**b**) 3-DG, and (**c**) 3-DGal are shown. All experiments were performed in triplicates. Significant differences between the samples with and without HSA are marked (* *p* < 0.05, ** *p* < 0.01, *** *p* < 0.001).

HSA was selected as the first compound of interest because of its high concentration in human serum [26] and the reduced thiol group of Cys-34, which represents the main source of free thiol in human plasma and is, therefore, essential for its antioxidant activity [27]. Moreover, HSA has several important physiological functions. Amongst those are the transport and binding of several molecules, such as fatty acids, hormones [28], and drugs [29] but also prodrug-activation through its esterase-like activity [30]. Previous studies have shown that 3,4-DGE is predominantly attacked by thiol groups [7,8]. This strengthens our hypothesis that HSA may be a principal reaction partner of 3,4-DGE in blood. Thus, physiological concentrations of HSA (40.0 mg/mL) were incubated with 3,4-DGE (165.0 µM) in supplemented phosphate buffered saline (*s*-PBS) containing calcium, magnesium, and the fasting plasma concentration of glucose at 37 °C for 48 h. Subsequently, non-bound 3,4-DGE was analyzed by ultrahigh-performance liquid chromatography coupled to a diode array detector (UHPLC-DAD). After 7 h, only 8.5% of the initial concentration was detected (vs. 83.4% in the control, *p* < 0.001). The concentration decreased further to 3.2% after 24 h (vs. 52.4% in the control, *p* < 0.001) and to 1.6% after 48 h (vs. 29.6% in the control, *p* < 0.001; Figure 1a). Thus, it can be concluded that HSA reacts quickly and efficiently with 3,4-DGE after its possible transition into the human blood circulation, which may lead to a quick decline of the 3,4-DGE concentration in plasma. This high reactivity of HSA with 3,4-DGE may explain why 3,4-DGE was no longer detectable in plasma after its incubation for 24 h in vitro [24].

**Scheme 1:**
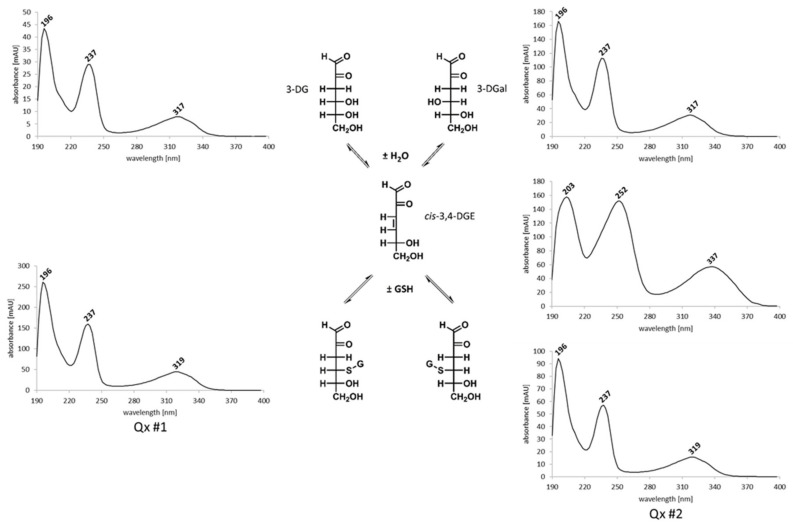
3,4-Dideoxyglucosone-3-ene (3,4-DGE) reacts with water or l-glutathione (GSH) via Michael addition. Samples containing 3,4-DGE (165.0 µM) and 91.0 µM GSH were incubated for 24 h at 37 °C, derivatized by *ortho*-phenylenediamine (*o*-PD) and analyzed by UHPLC-DAD. Quinoxaline UV-spectra (incl. values of the absorption maxima) of the formed reaction products are depicted next to their corresponding molecular structures. The abbreviations, Qx #1 and Qx #2, refer to the signals of the new quinoxalines shown in Figure 2 but have been assigned arbitrarily to the isomers.

The presence of HSA also reduced the interconversion of 3,4-DGE to 3-DG and 3-deoxygalactosone (3-DGal), which are both formed by its reaction with water (Scheme 1).

While the 3-DG concentration in HSA-free samples increased from 3.2 µM at incubation start (3-DG traces in synthesized 3,4-DGE) to 17.3 µM (*p* < 0.001) after 48 h, it rose from 3.1 to only 5.2 µM (*p* < 0.05) in the presence of HSA (Figure 1b). The same trend was observed for 3-DGal, a stereoisomer of 3-DG, whose concentration in HSA-free samples increased from 9.1 µM at the incubation start (3-DGal traces in synthesized 3,4-DGE) to 73.5 µM (*p* < 0.001) after 48 h. In the presence of HSA, however, the 3-DGal content increased only from 7.8 to 12.4 µM (*p* < 0.05; Figure 1c). It has been shown before that 3,4-DGE can be rehydrated to 3-DG and 3-DGal at 120 °C in glucose-free peritoneal dialysis fluids [31]. In the present experiments, this reaction was also observed under physiological conditions, particularly in the HSA-free solutions. Interestingly, this interconversion was highly affected by the presence of HSA because 3,4-DGE was partially removed from the solution by the protein. Previously, Tauer et al. incubated a single-chamber peritoneal dialysis fluid (pH 7.5) for three weeks at 37 °C with and without 1 mg/mL HSA. The presence of HSA did not influence the 3-DG concentration but led to an increased content of the AGE, imidazolone [32].

### 2.2. Effects of Glycation with 3,4-Dideoxyglucosone-3-ene on the Thiol Content, Ketoprofen-Binding, and Esterase-like Activity of Human Serum Albumin

The observed reaction of 3,4-DGE with HSA may lead to an efficient detoxification in vivo. On the other hand, 3,4-DGE may also form AGE adducts of HSA, which could impair its biological function. Further experiments were conducted to investigate if the reaction between both compounds may affect HSA functions.

HSA was incubated with 3,4-DGE for 7 h. At this time point, only 8.5% of the initial 3,4-DGE was detectable, indicating that the majority was bound to HSA. Subsequently, the protein was purified and subjected to several assays. First, Ellman’s assay assessed the amount of accessible thiol in the protein incubated with and without 3,4-DGE. Although each HSA molecule contains one free thiol group at the Cys-34, only a minor part is accessible to the Ellman’s reagent since it is situated in a pocket [33]. Validation of the assay showed good linearity (*R*^2^ = 0.9997) in the calibration range of 11–77 µM. The recovery was tested for two l-cysteine concentrations (24.1 µM and 36.7 µM), resulting in 97.2% and 92.9%, respectively. After 7 h of incubation with 3,4-DGE, a ratio of 0.064 ± 0.015 mol accessible thiols per mol HSA was determined, compared to 0.121 ± 0.011 mol accessible thiol per mol HSA in the control without 3,4-DGE; the difference was statistically significant (*p* < 0.01). These results confirm that the presence of 3,4-DGE leads to a considerable loss of reduced cysteine in HSA. Baraka-Vidot et al. incubated HSA for three weeks with varying glucose concentrations under nitrogen at 37 °C in PBS [34]. While they measured a ratio of 0.122 mol accessible thiol per mol HSA in the absence of glucose, the ratio dropped to 0.087 at the highest applied glucose concentration (500 mM) [34]. In comparison, our findings underline the higher reactivity of 3,4-DGE towards free thiol groups and its stronger glycating ability compared to glucose.

Additionally, we assessed how the reaction with 3,4-DGE influenced two pharmacologically relevant functions of HSA, namely the ketoprofen-binding and the esterase-like ability. The observable hydrolysis rates, k_obs_, for 20 µM HSA amounted to 1.17 × 10^−3^/s in the samples with 3,4-DGE and to 0.538 × 10^−3^/s in the samples without 3,4-DGE, but no statistically significant difference was observed. Therefore, the esterase-like activity was not influenced by 3,4-DGE. The ketoprofen-binding capacity was also not affected as indicated by the number of sites available for the binding of ketoprofen: 1.51 ± 0.02 in the glycated HSA and 1.49 ± 0.02 in the control, without significant difference. In contrast to expectations, a higher binding constant, K_A_, was observed for the glycated protein than for the control (3.26 × 10^6^ L/mol, compared to 2.89 × 10^6^ L/mol), although the difference was not statistically significant. Moreover, a closer look into the raw data revealed that the higher apparent binding rate was caused by a significantly lower (*p* < 0.05) basal fluorescence of the glycated HSA, while the decrease of fluorescence intensity did not statistically differ between both samples after the addition of ketoprofen. Therefore, we assumed that, in sum, the ketoprofen-binding capacity was not affected by 3,4-DGE. Arg-410 is found in the so called Sudlow Site II of HSA and is involved both in the drug-binding of ketoprofen-like drugs and in esterase-like activity of the protein [35]. Previous studies have shown that both glucose [34] and methylglyoxal [36] are able to glycate this amino acid residue, thereby impairing both functions. By comparison of these findings to the present results, we hypothesize that Arg-410 is not predominantly glycated by 3,4-DGE. Therefore, we rather expect a Michael addition of thiol groups because of its unsaturated dicarbonyl group than a reaction of the dicarbonyl group directly to arginine, which is supported by the reduced accessible thiol content after glycation. However, other reactive amino acid side chains, such as lysine, may be involved as well [7].

These findings indicate that the glycation of HSA by 3,4-DGE might result in a decrease of free cysteine residues, which possibly reduces its antioxidative property, while the tested pharmacologically relevant functions seem to be unaffected. Consequently, this reaction may represent a pathway that rather prevents further harmful reactions of 3,4-DGE than leading to HSA damage.

### 2.3. Reactivity of 3,4-Dideoxyglucosone-3-ene towards l-Glutathione

**Figure 2:**
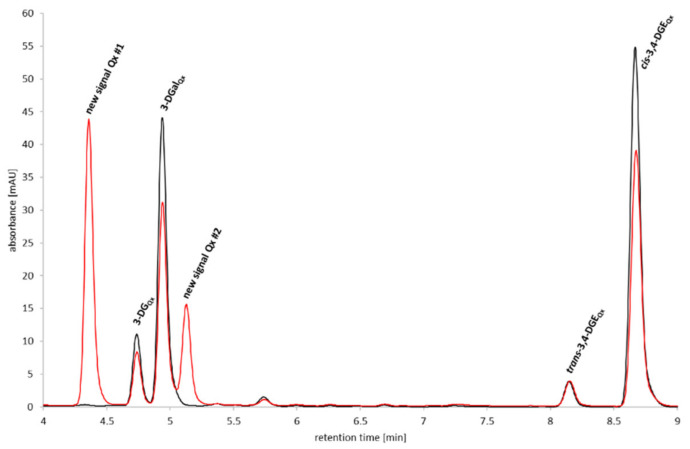
Incubation of 3,4-dideoxyglucosone-3-ene (3,4-DGE) with l-glutathione (GSH) leads to the formation of two new signals. 3,4-DGE (165.0 µM) was incubated either without (black curve) or with (red curve) 91.0 µM GSH at 37 °C for 24 h and analyzed by UHPLC-DAD at 316 nm after derivatization with *ortho*-phenylenediamine (*o*-PD). An excerpt of the chromatogram is shown; the identified compounds are labeled. The UV-spectra are depicted in Scheme 1; the characteristic quinoxaline-UV-spectra of the new signals, Qx #1 and Qx #2, indicate the presence of dicarbonyl compounds. Qx, quinoxaline.

Secondly, GSH was selected as a substrate for incubation with 3,4-DGE. Our experiments monitored if and how plasma concentrations of GSH (18.2 µM in healthy individuals [37]) reacted with 3,4-DGE (165 µM) under physiological conditions in *s*-PBS at 37 °C during incubation for 48 h. For the quantification of the remaining 3,4-DGE contents, the samples were derivatized with *ortho*-phenylenediamine (*o*-PD) and analyzed by UHPLC-DAD. Already, immediately after the addition of 3,4-DGE, the formation of two new signals at 4.36 min and 5.13 min could be observed, which eluted at similar retention times as 3-DG_Qx_ and 3-DGal_Qx_. To improve the quality of the UV-spectra, the GSH concentration was increased to 91.0 µM, thus enhancing the signal intensity (Figure 2). Both signals showed spectra that were typical for saturated quinoxaline structures (Scheme 1), indicating the formation of two saturated α-dicarbonyl compounds in the reaction mixture.

**Figure 3:**
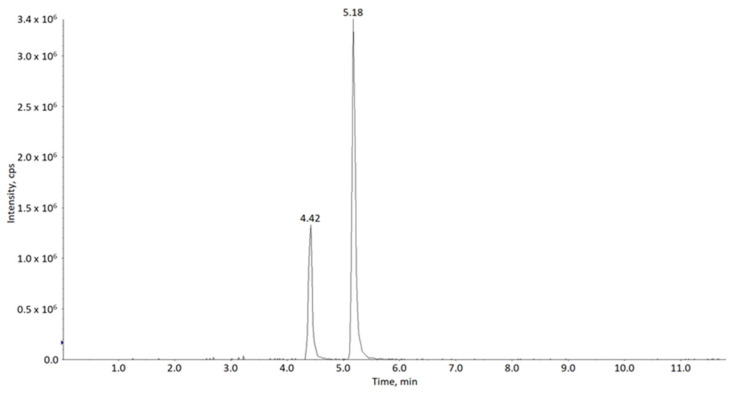
Incubation of 3,4-dideoxyglucosone-3-ene (3,4-DGE) with l-glutathione (GSH) results in two products with a *m/z* value of 524.3 Da. Total ion chromatogram of an enhanced product ion scan (EPI) for the *m/z* value of 524.3 Da for the model solution containing 165.0 µM 3,4-DGE and 91.0 µM GSH after incubation at 37 °C for 1 h after derivatization with *ortho*-phenylenediamine (*o*-PD).

The signal intensity of 3,4-DGE decreased at the same time (Figure 2). Remarkably, the concentration of its *cis*-isomer was mainly affected. To elucidate the structures of the newly formed products, the sample was analyzed by ultrahigh-performance liquid chromatography coupled to tandem mass spectrometry (UHPLC–MS/MS) in enhanced mass spectrometry scan (EMS) mode. First, specific *m/z* values at the retention times of the new signals were searched. At both retention times, the *m/z*-value, 524.3 Da, could be detected and extracted, indicating that it is specific to the retention time of the two new signals. To gain further insight into the molecular structure of the two compounds, enhanced product ion (EPI) spectra were recorded for the parent ion, *m/z* 524.3 Da (Figure 3).

**Scheme 2:**
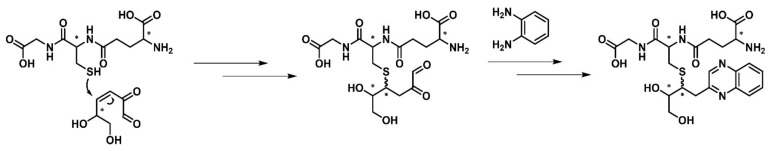
3,4-Dideoxyglucosone-3-ene (3,4-DGE) and l-glutathione (GSH) react via Michael addition to a monoadduct, which is subsequently derivatized to a quinoxaline. Simplified reaction mechanism for the reaction of 3,4-DGE with GSH during incubation under physiological conditions as well as for the formation of the quinoxaline derivative after reaction with *ortho*-phenylenediamine (*o*-PD). Stereocenters are depicted with asterisks.

**Figure 4:**
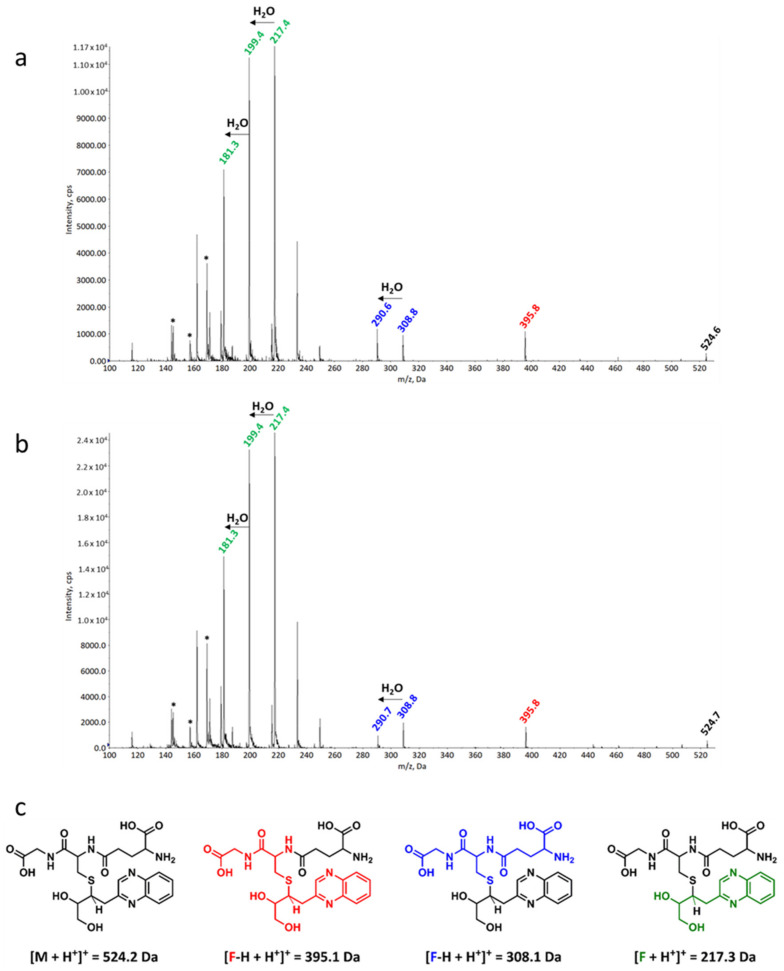
Signals identified as quinoxaline derivatives of a 3,4-dideoxyglucosone-3-ene – l-glutathione (3,4-DGE-GSH) monoadduct. Enhanced product ion (EPI) spectra gained through fragmentation of (**a**) the parent ion (*m/z* 524.6 Da) at a retention time of 4.42 min and (**b**) the parent ion (*m/z* 524.7 Da) at 5.18 min are shown, including experimental *m/z* values of the identified fragments (as well as after the loss of water molecule(s)) above their respective signal. (**c**) The proposed molecular structures of the adduct and its fragments are depicted together with their theoretical *m/z* values. The hyphen “-” between the fragment (F) and the hydrogen (H) symbolizes a bond between these. Fragments derived from the 3,4-DGE_Qx_-backbone are labeled with asterisks. Stereocenters are omitted for clarity.

The EPI chromatograms supported the findings from the EMS and resulted in two signals at 4.42 min and 5.18 min. The two respective product ion spectra gained through fragmentation of the parent ion were nearly identical to each other (Figure 4a,b). The signals of the parent ions, [M+H]^+^, at 524.6/524.7 Da indicate that GSH forms two monoadducts with 3,4-DGE, which are subsequently derivatized to the respective quinoxalines. This assignment was confirmed by the fragmentation patterns, which were identical for both products. Three main fragments were identified (*m/z* 395.8 Da, 308.8 Da, and 217.4 Da) together with the corresponding fragments after a loss of water. Structures for these fragments are proposed in Figure 4c and are in good accordance with typical fragmentation patterns reported for GSH-xenobiotic adducts [38]. These fragments support the proposed mechanism that the thiol group of GSH attacks the conjugated double bond of 3,4-DGE via a Michael addition from two possible sites, forming two stereoisomers (see Scheme 2).

**Figure 5:**
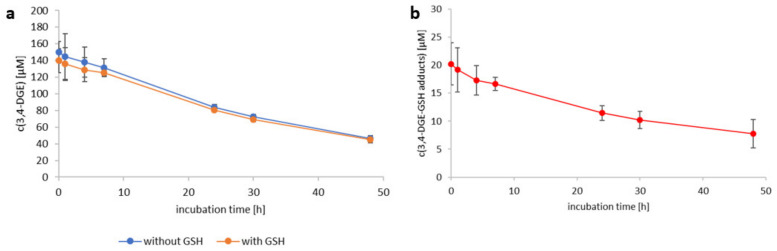
l-Glutathione (GSH) leads to an immediate formation of monoadducts with 3,4-dideoxyglucosone-3-ene (3,4-DGE). (**a**) Time-course curves are shown for the concentrations (in µM, mean ± SD; 3,4-DGE start concentration as analyzed 150.0 µM) of 3,4-DGE during 48 h of incubation at 37 °C either in absence (blue curve) or presence (orange curve) of GSH (18.2 µM). (**b**) The time-course curve for the concentration of the formed monoadducts is shown in red. The interconversion of 3,4-DGE to 3-deoxyglucosone (3-DG) and 3-deoxygalactosone (3-DGal) was not affected. All experiments were performed in triplicates; the areas of the two 3,4-DGE–GSH monoadducts were summed up. The differences between the samples with and without GSH are not significant.

The nucleophilic attack of water on the double bond of 3,4-DGE, leading to the rehydration product 3-DG or its stereoisomer 3-DGal, has been demonstrated before [31]. Previously, Yamamoto et al. have reported that 3,4-DGE can react with GSH, forming an adduct [12]. Already after incubating human peritoneal mesothelial cells with 3,4-DGE for one hour, the total intracellular GSH content was depleted. Moreover, the formed adduct has been identified via liquid chromatography-electrospray ionization (ESI)-MS without previous derivatization [12]. The presently applied chromatographic method was able to separate the derivatized two stereoisomers, 3-DG and 3-DGal, and revealed the formation of two 3,4-DGE–GSH adducts. Furthermore, their UV-spectra, which are typical for quinoxalines containing saturated side chains, strongly differed from the UV-spectrum of 3,4-DGE_Qx_ (Scheme 1). In 3,4-DGE_Qx_, the double bond is still intact and leads, therefore, to a different absorption behavior. This observation also suggests that the original double bond was attacked by GSH. Together with Yamamoto’s findings, our results underline that this reaction and the resulting depletion of GSH may be one reason for the well-known cytotoxicity of 3,4-DGE towards several cell types in vitro. The proposed adducts were quantified using the calibration curve of 3-DG_Qx_ because those compounds possess the same chromophore (Scheme 1). Directly after adding 3,4-DGE to physiological concentrations of 18.2 µM GSH, about 20.2 ± 3.8 µM of adducts were formed (Figure 5b). This result indicates that GSH reacts immediately and quantitatively to the detected monoadducts. Over time, the adduct concentration decreased. After 48 h, only 38.5% of the original concentration was still present. It is known that the formation of GSH adducts can be reversible under physiological conditions [39]. In accordance with the immediate formation of about 20 µM adducts, the 3,4-DGE concentration in the presence of GSH dropped to 139.8 ± 14.5 µM, compared to 150.0 ± 13.0 µM in the absence of GSH. Since this decrease was low compared to the initial 3,4-DGE concentration, this difference was not significant. Over time, the 3,4-DGE levels equaled in samples with and without GSH, which could be caused by the release of free 3,4-DGE from the adducts (Figure 5a). Hence, the interconversion to 3-DG and 3-DGal was not influenced.

Since 3,4-DGE reacts immediately and quantitatively with GSH, it seems plausible that the highly reactive 3,4-DGE might affect numerous functions of GSH. This tripeptide, which is present in most mammals, counteracts oxidative stress due to its reduced thiol moiety [40] and conjugates strong electrophiles, preventing their reaction with functional biomolecules in vivo. The reaction named *glutathione conjugation* can either take place non-enzymatically or under support of glutathione-S-transferase [41]. Additionally, GSH acts as a cofactor in the glyoxalase system that is responsible for clearing compounds, such as methylglyoxal and glyoxal [42]. The conjugation of GSH to 3,4-DGE may, thus, enhance oxidative stress and reduce the clearance of other toxic electrophiles. Glyoxalase 1, which uses GSH as a cofactor to metabolize compounds, such as glyoxal, might also be affected, leading to higher systemic glycation. Moreover, the observed conjugation of GSH by 3,4-DGE may influence the reduction of H_2_O_2_, which is catalyzed by glutathione peroxidase, requiring GSH as a cofactor. Thus, oxidative stress may be further enhanced by the formation of ROS [40]. Although no significant difference was observed in our experiments between 3,4-DGE concentrations in the presence or absence of GSH, we assume that plasma GSH might be sufficient to successfully degrade 3,4-DGE formed in vivo because its physiological concentrations are probably much lower than the contents in the present test setup. Thus, the non-enzymatic GSH-conjugation of 3,4-DGE might represent a pathway for its detoxification. It is well known that GSH-conjugates are subsequently converted to mercapturic acids and afterwards excreted [41]. Overall, the observations for GSH, as well as the results of the present experiments with HSA, once more substantiate that 3,4-DGE is often attacked on its conjugated double bond, especially by thiol-containing compounds.

### 2.4. Reactivity of 3,4-Dideoxyglucosone-3-ene towards Human Immunoglobulin G

**Figure 6:**
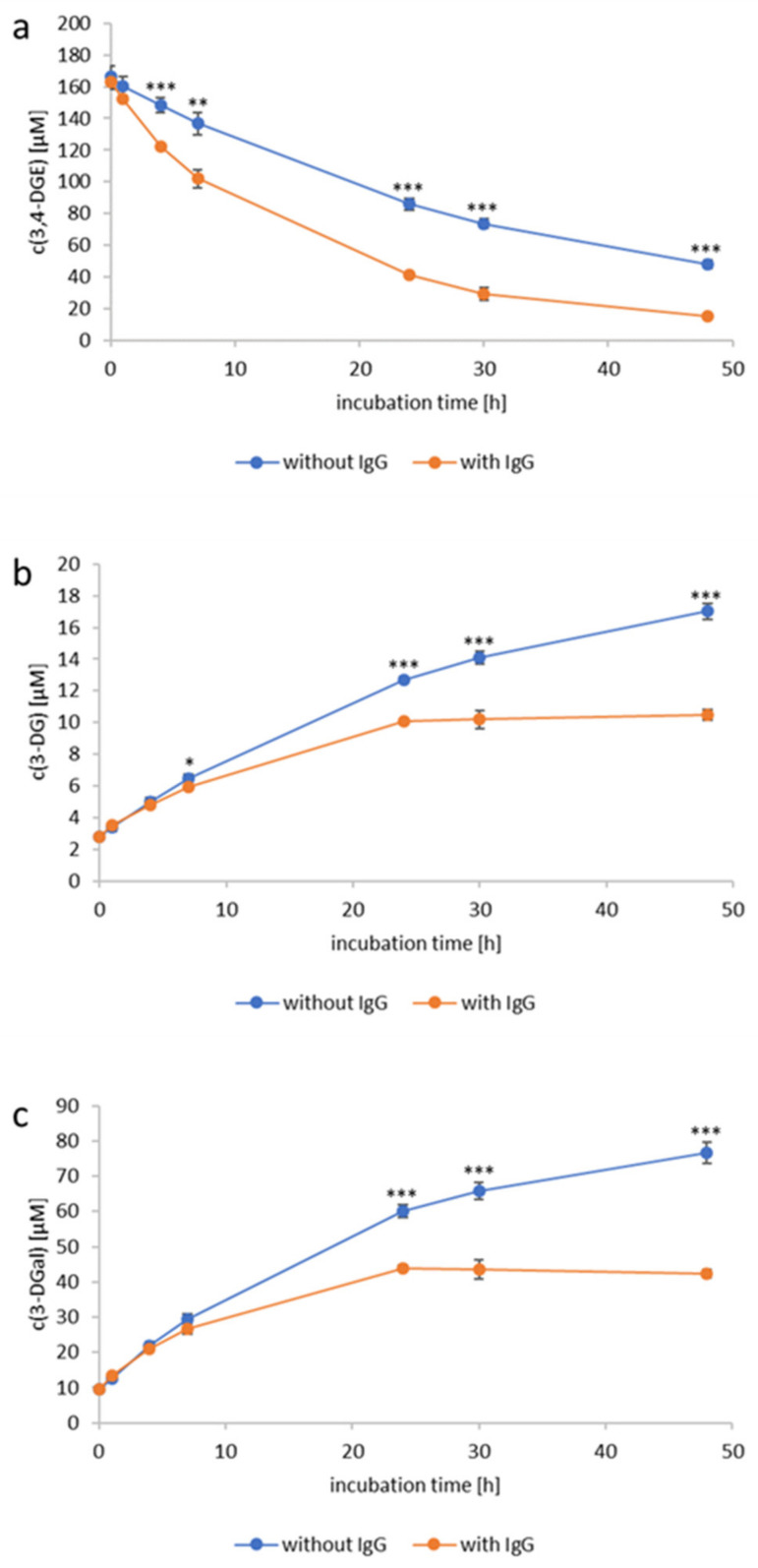
Human immunoglobulin G (IgG) partially depletes3,4-dideoxyglucosone-3-ene (3,4-DGE) from the model solution and, therefore, disables its interconversion to 3-deoxyglucosone (3-DG) and 3-deoxygalactosone (3-DGal). 3,4-DGE (166.2 µM, containing 2.8 µM 3-DG and 9.5 µM 3-DGal) was incubated for 48 h at 37 °C in the absence (blue curve) or presence (orange curve) of IgG (11.5 mg/mL). The time-course curves for the concentrations (in µM, mean ± SD) of (**a**) 3,4-DGE, (**b**) 3-DG, and (**c**) 3-DGal are shown. All experiments were performed in triplicates. Significant differences between the samples with and without IgG are marked (* *p* < 0.05, ** *p* < 0.01, *** *p* < 0.001).

As a third possible target for 3,4-DGE, human IgG was evaluated because of its high abundance in human blood and its importance for the immune system. Consisting of four subclasses, IgG reaches serum concentrations in adults of about 11.85 mg/mL. It can bind antigens, such as proteins, polysaccharides, allergens, and toxins, and, thus, stimulate immune responses [43]. In the present study, IgG (11.5 mg/mL) was incubated with 3,4-DGE (165 µM) as described for HSA. After 7 h, 62.6% of the initial 3,4-DGE contents was left (vs. 82.2% in the control, *p* < 0.01), further decreasing to 25.2% (vs. 51.7% in the control, *p* < 0.001) after 24 h and to 9.3% (vs. 28.9% in the control, *p* < 0.001) after 48 h (Figure 6a). The interconversion of 3,4-DGE to 3-DG and 3-DGal was affected as well but not as strongly as in the presence of HSA. While the 3-DG concentration in IgG-free samples increased from 2.8 µM at the incubation start (3-DG traces in synthesized 3,4-DGE) to 17.0 µM (*p* < 0.001) after 48 h, it increased only from 2.8 to 10.5 µM (*p* < 0.001) in the presence of IgG (Figure 6b). The same trend was observed for 3-DGal, whose concentration in IgG-free samples rose from 9.5 µM at the incubation start (3-DGal traces in synthesized 3,4-DGE) to 76.7 µM (*p* < 0.001) after 48 h but only from 9.6 to 42.4 µM (*p* < 0.001) in the presence of IgG (Figure 6c).

The present findings show that 3,4-DGE is indeed able to react with IgG, although not as strongly as with HSA, which may be due to the fact that IgG contains about 60% less free thiols than HSA [44]. Additionally, the tested IgG concentrations were lower compared to HSA, reflecting the respective plasma concentrations in vivo. Glycation of IgG is known to occur in vivo, especially in diabetic patients, where it may represent a promotive factor for concomitant inflammations and impaired immune response [45]. Some in vitro glycation studies have shown effects on antigen-antibody affinity, such as in glucose-glycated mouse IgG [46], but others have reported that the glycation of therapeutic IgG by glucose has not influenced its potency [47]. AGE-modified IgG detected in rheumatoid arthritis patients seems to have induced an immune response that led to the formation of further antibodies against this AGE-modified IgG [48]. Kato et al. have found immunosuppressive effects of 3,4-DGE, namely in mice suffering from induced delayed-type hypersensitivity and arthritis but also on murine T cell proliferation, antibody, and interleukin-1 production in vitro [16]. Whether the direct glycation of IgG by 3,4-DGE might have any effect on the immune response remains to be elucidated. As observed in the case of HSA, this reaction might also be regarded as detoxification of 3,4-DGE.

### 2.5. Reactivity of 3,4-Dideoxyglucosone-3-ene towards Human Collagen Type IV

**Figure 7:**
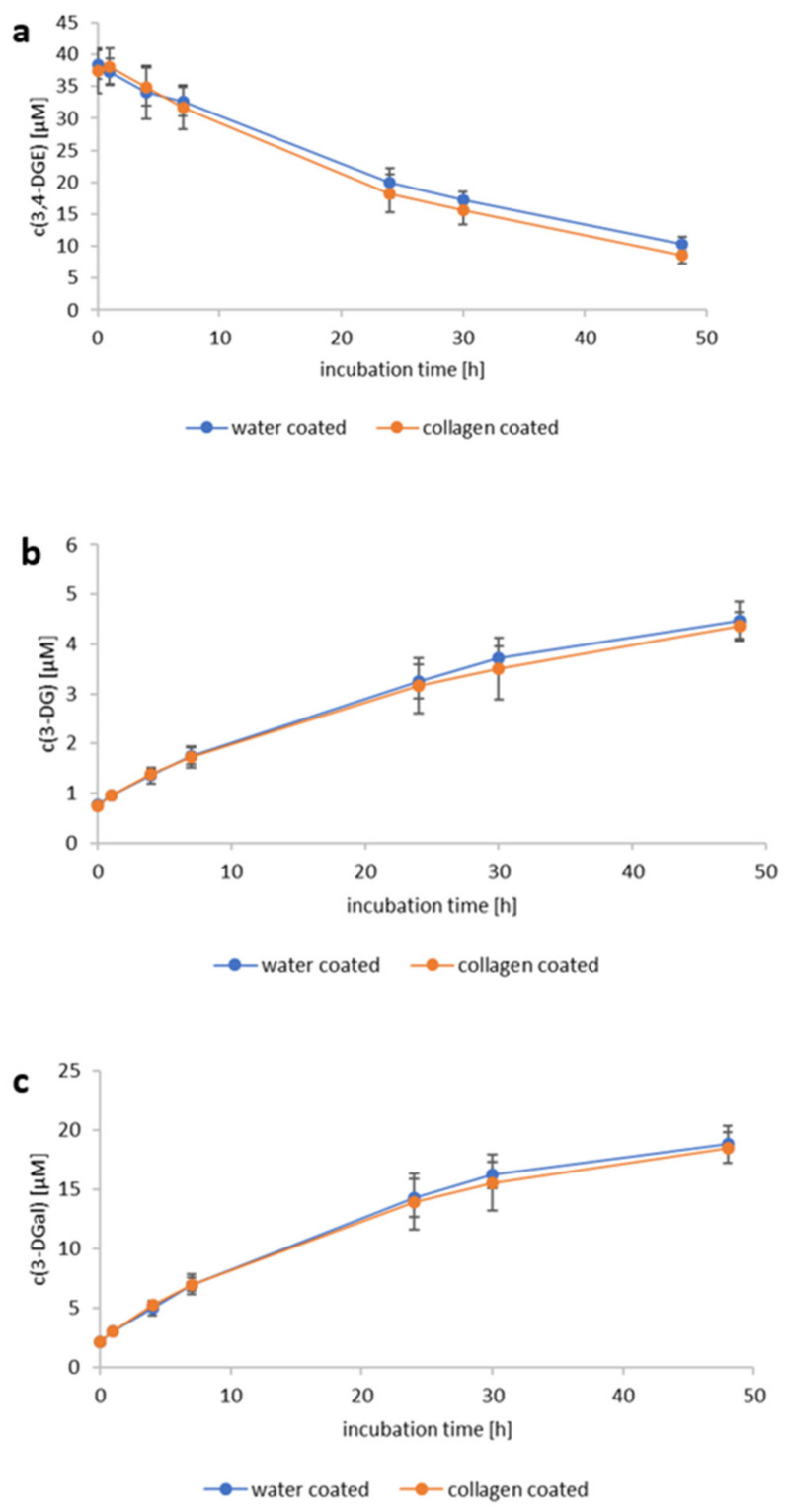
Human placenta-derived collagen does not interact with 3,4-dideoxyglucosone-3-ene (3,4-DGE) and does not affect its interconversion to 3-deoxyglucosone (3-DG) and 3-deoxygalactosone (3-DGal). 3,4-DGE (38.4 µM, containing 0.8 µM 3-DG and 2.1 µM 3-DGal) was incubated for 48 h at 37 °C in non-coated (blue curve) or in collagen-coated tubes (orange curve). The time-course curves for the concentrations (in µM, mean ± SD) of (**a**) 3,4-DGE, (**b**) 3-DG, and (**c**) 3-DGal are shown. All experiments were performed in triplicates. The differences between the samples incubated in coated and non-coated tubes are not significant.

Collagen type IV is the main collagen type found in basement membranes [49] and represents one of the principal components of blood vessels that is next to the endothelium and in proximal contact with the blood stream. Therefore, we investigated a possible reaction between this collagen type and 3,4-DGE. For this purpose, Eppendorf tubes (Hamburg, Germany) were coated with reconstituted collagen to mimic vessels and subsequently incubated with and without 3,4-DGE in *s*-PBS at 37 °C for 48 h. Because of the comparably low protein content in the tube during incubation, a lower 3,4-DGE concentration of 41.3 µM was applied so that slighter differences between the analyte concentrations in the coated and non-coated tubes could be detected. In fact, no significant differences could be found, and the 3,4-DGE degradation as well as its interconversion to 3-DG and 3-DGal followed the same trends, as described for the substrate-free solutions (Figure 7). Previously, Tauer et al. have observed that collagen had no effect on 3-DG and glyoxal concentrations after incubation of glucose-based peritoneal dialysis fluids in collagen-coated wells (type not specified) for six hours [32]. On the other hand, glycation of collagen has been reported, e.g., in collagen derived from rat-tail tendon incubated with millimolar concentrations of glucose [50] or ribose [51]. It seems, therefore, that glycation of collagen is highly dependent on both the collagen type and glycating agent. Moreover, the typical triple-helical domains of collagen and its aggregation to supramolecular structures [52] might contribute to the limited reactivity: Since part of the amino acid side chains are aligned towards the interior of the helices and assemblies, those are not as readily available for reaction with 3,4-DGE as the amino acids on the surface.

While we selected physiological concentrations of the three substrates, HSA, GSH, and IgG, a relatively high 3,4-DGE concentration was applied in all experiments so that the 3,4-DGE levels remained above the limit of quantification (0.40 µM) during the whole incubation course. Thus, it was possible to observe changes in the analyte content caused by the presence of the selected substrates. At the same time, the high concentration of 3,4-DGE led to a well-detectable effect on the free-thiol content in the case of HSA. Medicinal products, however, contain less 3,4-DGE. The 3,4-DGE possibly formed in vivo would be in the nanomolar range [14] but would be continuously released from the 3-DG pool in blood. Therefore, it can be expected that the components of the human blood circulation reduce the 3,4-DGE concentrations even stronger in vivo. Further studies are now required to investigate the effects of 3,4-DGE applied in lower concentrations.

## 3. Materials and Methods

### 3.1. Reagents

Ultrapure water (Milli-Q^®^ Reference A+ system, Merck Millipore, Darmstadt, Germany) was used for the preparation of all solutions and mobile phases. Methanol (Fisher Scientific, Schwerte, Germany), formic acid (VWR International, Ismaning, Germany), and ammonium formate (Sigma Aldrich, Taufkirchen, Germany) for UHPLC-DAD and -MS/MS analysis were of LC-MS grade purity. *N*-2-Hydroxyethylpiperazine-*N*′-2-ethane sulphonic acid (HEPES; purity ≥ 99.5 %) was purchased from Carl Roth (Karlsruhe, Germany), hydrogen chloride and sodium hydroxide (pro analysi) from Grüssing (Filsum, Germany). *o*-PD (≥98%), 2,3-dimethylquinoxaline (97%), potassium chloride (≥99.0%), calcium chloride dihydrate (≥99.5%), glucose (≥99.5%), reduced GSH (≥98%), IgG from human serum (≥95%), collagen from human placenta (Bornstein and Traub type IV), 5,5′-dithio-*bis*-(2-nitrobenzoic acid) (99%, DTNB), l-cysteine (≥98%, from non-animal source), ketoprofen (≥98%), and *p*-nitrophenyl-acetate (≥98%, *p*-NPA) were obtained from Sigma Aldrich (Taufkirchen, Germany), and potassium phosphate (analytical reagent grade) from Sigma-Aldrich Laborchemikalien (Seelze, Germany). Sodium chloride, disodium hydrogen phosphate dihydrate, and magnesium chloride hexahydrate (all three pro analysi) were purchased from Merck (Darmstadt, Germany). The 3,4-DGE and 3-DGal were synthesized, as described previously [20,53], whereas 3-DG was obtained from Chemos (Altdorf, Germany). The 3,4-DGE solution contained traces of 3-DG and 3-DGal. HSA (≥95%, non-denatured and salt-free) was purchased from EMD Millipore (Burlington, MA, USA) and the Pierce BCA Protein assay kit from Fisher Scientific (Schwerte, Germany).

### 3.2. Coating of Eppendorf Tubes with Collagen

Ultrapure water (10 mL) was added for the reconstitution of 10 mg of human placenta-derived collagen (Bornstein and Traub type IV) to obtain a concentration of 1 mg/mL, as used by Tauer et al. [32]. The mixture was shaken gently for 5 h at 3 °C until the solid was dissolved. Aliquots were kept at −20 °C until use. After thawing at 5 °C, the solution was stored in Eppendorf tubes (Hamburg, Germany) overnight at 5 °C. Directly before the 3,4-DGE test solution was added to the coated tubes, the opened tubes were put in a heating block at 37 °C while the solvent was removed under nitrogen stream.

### 3.3. Incubations of 3,4-Dideoxyglucosone-3-ene with Components of the Human Blood Circulatory System

For all incubations, *s*-PBS (pH 7.4; 137 mM sodium chloride, 2.7 mM potassium chloride, 10 mM disodium hydrogen phosphate dihydrate, 1.8 mM potassium phosphate, 1 mM calcium chloride dihydrate, 0.5 mM magnesium chloride hexahydrate) was used, including 5.5 mM glucose, which is the mean fasting plasma level of males [54]. For washing, purification, and assays, glucose-free *s*-PBS was applied.

The 3,4-DGE (165.0 µM) in *s*-PBS was incubated with physiological concentrations of HSA (40.0 mg/mL), IgG (11.5 mg/mL), or GSH (18.2 µM), respectively. For the experiments in collagen-coated Eppendorf tubes, 41.3 µM 3,4-DGE was added. The samples were incubated in a dry heating block (Thermomixer comfort; Eppendorf, Hamburg, Germany) at 37 °C and 300 rpm for up to 48 h. The incubation was stopped by cooling the tubes in a water bath at 4 °C for 1 min. Controls without the components of the human blood circulatory system were incubated and treated analogously. Except for the GSH-containing samples and their respective controls, all samples underwent ultrafiltration directly after incubation to prevent further reactions and clogging of the analytical columns during analysis of the filtrates. For this purpose, centrifugal filters (modified polyethersulfone (PES), molecular weight cut-off (MWCO) 10 kDa, 500 µL capacity, low protein binding; VWR International, Ismaning, Germany) were pre-wetted twice with ultrapure water for 20 min at 13,910× *g*. Afterwards, the sample was added and centrifuged for 23 min at 13,910× *g*. The used filters were washed three times with 90 µL of glucose-free *s*-PBS to collect all traces of non-bound GDPs. All filtrates were then stored at −20 °C until analysis. All incubations were performed in independent triplicates.

### 3.4. Quantitative UHPLC-DAD Analysis of Dicarbonyl Compounds and l-Glutathione Adducts

The thawed filtrates were derivatized and analyzed by UHPLC-DAD, as described before [53]. Following this method, 80 µL of sample containing α--dicarbonyls were reacted with 20 µL of derivatizing reagent containing *o*-PD and 2,3-dimethylquinoxaline, as the internal standard. After at least 2 h, the formed quinoxalines were subsequently separated, applying a multi-step gradient, consisting of ammonium formate buffer (pH 2.8) and methanol on an ACQUITY UPLC^®^ BEH phenyl column (2.1 × 100 mm, 1.7 µm particle size; Waters, Eschborn, Germany) equipped with a pre-column of the same material, detected by DAD at 316 nm (3-DG_Qx_ and 3-DGal_Qx_) and 335 nm (3,4-DGE_Qx_), and quantified via external calibrations. The applied UHPLC-DAD system (Ultimate 3000RS, Thermo Fisher Scientific, Dreieich, Germany) comprised a pump with degasser, autosampler, column oven, and DAD. The software, Chromeleon 6.80 (Thermo Fisher Scientific, Dreieich, Germany), was used for system control and data analysis. The areas of the two 3,4-DGE–GSH monoadducts were summed up and quantified via the 3-DG calibration curve because of their similar absorption behavior.

### 3.5. Qualitative UHPLC-DAD and -MS/MS Analysis of the 3,4-Dideoxyglucosone-3-ene-l-Glutathione Adducts

The 3,4-DGE (165.0 µM) in *s*-PBS was incubated with 91.0 µM GSH for 1 h at 37 °C. For UHPLC-DAD analysis, the undiluted solution was derivatized directly and analyzed, as described above. For the UHPLC–MS/MS analysis, the solution was diluted 1:4 in glucose-free *s*-PBS before derivatization. The UHPLC-model described in 3.4 for the UHPLC-DAD analysis was applied for chromatographic separation but was coupled to an API 4000 QTrap mass spectrometer (AB Sciex, Darmstadt, Germany) with an ESI source. The Sciex software Analyst 1.6.2 was used for system control and data analysis. Parameters were applied, as reported previously [53], with slight variations to find the newly formed adducts; an EMS scan from 100 to 550 Da was performed with a reduced declustering potential of 60 V and a collision energy of 10 V. To identify and elucidate the structures of the two compounds, fragmentation in EPI mode collected fragments between 100 and 530 Da. Therefore, the *m/z*-value of 524.3 Da, which was detected during EMS, was selected as the parent ion, the declustering potential was reduced to 20 V, and a collision energy of 30 V was chosen without application of a collision-energy spread.

### 3.6. Reaction of 3,4-Dideoxyglucosone-3-ene with Human Serum Albumin for Functional Assays

HSA was incubated with 3,4-DGE for seven hours, as described in Section 3.3. A control without 3,4-DGE was incubated in the same way. Subsequently, the samples were ultrafiltrated to remove non-bound traces of 3,4-DGE and glucose. For this purpose, centrifugal filters (Amicon Ultra—0.5 mL, Ultracel—10 kDa MWCO, regenerated cellulose; Merck Millipore, County Cork, Ireland) were pre-wetted once with glucose-free *s*-PBS for 30 min at 13,910× *g*. Afterwards, the sample was added and centrifuged for 30 min at 13,910× *g*, and the protein was washed three times with glucose-free *s*-PBS. For reconstitution, 100 µL of glucose-free *s*-PBS was added to the purified protein, the filter was turned upside down, and the protein was collected in a new Eppendorf tube (Hamburg, Germany) by centrifugation at 1000× *g* for 10 min. To collect all protein traces, the turned filter was washed two times with 165 µL of glucose-free *s*-PBS. All protein-containing solutions were combined (hereinafter referred to as *purified protein*) and stored at −80 °C until use. All incubations and the following assays were performed in independent triplicates. Protein concentrations were determined by Pierce BCA Protein assay (Fisher Scientific, Schwerte, Germany).

### 3.7. Analysis of Thiol Content, Ketoprofen-Binding, and Esterase-like Ability of Modified Human Serum Albumin

The thiol content was determined by Ellman’s assay, as described before [55,56], with minor changes. Presently, the purified protein was heated for 1 h at 37 °C before the addition of DTNB. Afterwards, the well plate was incubated in the dark for further 30 min at 37 °C. After cooling to room temperature, the absorbance was read at 412 nm. l-Cysteine was used as the standard, and two different concentration levels of HSA, namely 125 µM and 250 µM, were analyzed.

The ketoprofen-binding capacity and esterase-like activity towards *p*-NPA were assessed, as reported previously by Baraka-Vidot et al. [34]. The ketoprofen-binding assay was performed with a FP-6200 spectrofluorometer (Jasco Deutschland, Pfungstadt, Germany). All photometric measurements were made in sterile 96-well cell culture plates (Greiner Bio-One, Kremsmünster, Austria), employing a µQuant microplate reader (Biotek, Bad Friedrichshall, Germany). When incubation at 37 °C was required, the well plates were incubated in a temperature-controlled incubator shaker (Innova 42; New Brunswick Scientific, Edison, NJ, USA). Each sample was measured three times (technical replicates).

### 3.8. Statistics

Two-sided, unpaired student’s t-tests were performed to show significant differences between the samples with and without the biological substrate and between the samples with and without 3,4-DGE, respectively. Two-sided, paired student’s *t*-tests were performed to show significant differences between different time points of one incubation. Excel 2016 was used for all statistical evaluations. Significant differences were marked as * *p* < 0.05, ** *p* < 0.01, and *** *p* < 0.001.

## 4. Conclusions

With the goal to model the reactivity and stability of 3,4-DGE in the human blood circulation, 3,4-DGE was incubated under physiological conditions with the main reactive serum constituents. HSA, GSH, and IgG reacted readily with 3,4-DGE, leading to a quick decrease of the 3,4-DGE concentrations. These reactions could scavenge 3,4-DGE and, thus, contribute to its clearance and detoxification. On the other hand, the reaction of 3,4-DGE with HSA, GSH, and IgG may also lead to a loss of protein and GSH function. Particularly, the blockage of free-thiol groups in HSA and GSH may reduce protection against oxidative and electrophilic stress. In addition, the modification of IgG by 3,4-DGE may impair its immune function. Furthermore, the incubation with collagen type IV suggests that blood vessels are not strongly affected by 3,4-DGE. Two stereoisomeric 3,4-DGE adducts of GSH were detected, whose structures were assigned to diastereomers of the Michael adducts. Because of its high reactivity, the presence of 3,4-DGE in vivo and, consequently, its physiological relevance is difficult to determine. The identified GSH adducts or the corresponding cysteine adducts as probable metabolites may be used as stable markers for systemic 3,4-DGE exposure. Additionally, the compounds may help to elucidate the transformation of 3-DG into 3,4-DGE in vivo and its relevance in diabetic patients.

## Data Availability

Data supporting the conclusions of this study are available upon request from the authors.
